# Comparative proteomics analysis of adult *Haemonchus contortus* isolates from *Ovis ammon*

**DOI:** 10.3389/fcimb.2023.1087210

**Published:** 2023-03-16

**Authors:** Gongzhen Liu, Qing Liu, Zhaoqing Han, Peikun Wang, Yanshen Li

**Affiliations:** ^1^ College of Agriculture and Forestry, Linyi University, Linyi, Shandong, China; ^2^ Jinan Park Development Service Center, Jinan, Shandong, China; ^3^ Department of Marine Product Quality and Safety Inspection Key Laboratory, Yantai University, Yantai, Shandong, China

**Keywords:** *Haemonchus contortus (H. contortus)*, differentially expressed proteins (DEPs), Kyoto Encyclopedia of Genes and Genomes (KEGG), biological processes, label-free, *Ovis ammon (O. ammon)*, mouflon, proteomics

## Abstract

*Haemonchus contortus* is an important parasite that causes disease that seriously endangers ruminant animals cattle, sheep, goat, and camel. Here, we compared the proeomics analysis of three adult *Haemonchus contortus* isolates from mouflons (*Ovis ammon*). A total of 1,299 adult worm proteins were identified, and 461 proteins were quantified, of which 82 (108), 83 (97), and 97 (86) significantly upregulated (downregulated) differentially expressed proteins (DEPs) were detected among pairwise comparisons (1-vs.-3, 2-vs.-3, and 2-vs.-1). Liquid chromatography–tandem mass spectrometry (LC−MS/MS) and bioinformatic analysis indicated that these DEPs are mainly concentrated in cellular composition, molecular function, biological function, and catabolism pathways. In addition, Gene Ontology (GO) classification and Kyoto Encyclopedia of Genes and Genomes (KEGG) enrichment analyses were carried out to screen the DEPs. The main biological processes involved were nucleotide, nucleotide phosphate, ribonucleotide, purine-containing compound, purine ribonucleotide, single-organism, oxoacid, organic, carboxylic, oxoacid metabolic processes and single-organism catabolic processes. The majority of KEGG pathways were found to be related to metabolic pathways, biosynthesis of secondary metabolites, biosynthesis of antibiotics, carbon metabolism, and microbial metabolism in diverse environments. Moreover, we also found differences in the expression of some important or novel regulatory proteases, such as serine hydroxymethyl transferase (SHMT), dihydrolipoyl dehydrogenase (DLD), and transket pyr domain-containing protein (TKPD). In summary, label-free proteomic analysis of adult *H. contortus* worms displayed significant differences in three different individual isolates, which helps to improve our understanding of the growth and metabolic mechanisms of *H. contortus* in different individuals and relative natural environments and provides novel drug targets for the treatment of parasitic diseases.

## Introduction

*Haemonchus contortus* is one of the pathogenic nematodes of ruminant parasites in cattle, sheep, and goats (occasionally seen in the small intestine) (Aebersold and Mann, 2016; Ang et al., 2011), causing incalculable economic losses to most developed and developing countries and regions (Muchiut et al., 2018; Besier et al., 2016a). With the growth and reproductive capacity of animals infected with parasites, the production of meat and milk is obviously reduced, leading to inestimable economic losses in animal husbandry at home and abroad. *H. contortus* mainly feeds on the blood of infected animals, and the clinical symptoms often include loss of appetite, roughness, diarrhea, weight decline, abomasum mucosa damage, severe anemia, extreme thinning, and even death (Akkari et al., 2013; Wang et al., 2017). The severity of haemonchosis depends on a variety of factors, including the number of parasites, age, breed, nutrition, immune status, and environmental conditions, such as climate and vegetation. Currently, the control of *H. contortus* still depends on the use of anthelmintic drugs (Kotze and Prichard, 2016; Muchiut et al., 2018). However, the pressure of drug selection has caused serious drug resistance problems worldwide, and with the continuous excessive use and abuse of anthelmintics, the problem of drug resistance continues to intensify and has become another urgent problem of international prevention and control of parasitic disease.

As the executors of biological functions, proteins play a crucial role in maintaining the physiological activities of various organisms (Monti et al., 2019). Proteomics is an important life science field after genomics, including biological function, cellular positioning, and interaction proteins (Aebersold and Mann, 2016). It can elaborate the life process of organisms intuitively and reveal the law of life activities of organisms. Currently, proteomic technology based on liquid chromatography (LC) combined with tandem mass spectrometry (MS/MS) analysis has been widely used in proteomic research. The common methods are mainly divided into relative and absolute quantification, including stable isotope labeling with amino acids in cell culture (SILAC) and isobaric tags for relative and absolute quantitation (iTRAQ) technology (Yamini et al., 2022). iTRAQ enables simultaneous qualitative and quantitative analyses of proteins in four or eight different samples and is a high-throughput screening technique commonly used in proteomic research on parasitic diseases (Zhai et al., 2018; He et al., 2020). Compared with SILAC and iTRAQ technology, one more deep coverage in the high-throughput data-independent acquisition (DIA) method was applied in proteomic analysis, which can acquire a larger number of proteins than those of previous protein identification methods (Demichev et al., 2020).

In parasitological studies, most proteomic analyses are used to understand the biological characteristics of eggs or adult parasites, explore the protein profiles at different developmental stages, and analyze the protein differences at different stages or at the same stage (Di Maggio et al., 2022; Li et al., 2021; Wang et al., 2021; Wang et al., 2020; Hu et al., 2018; Wang et al., 2018). There are several research reports on the proteomics of *H. contortus* and its host animals. An LC−MS/MS-based approach was used to analyze 2,487 unique *H. contortus* proteins in different developmental stages/sexes with high confidence, which provided important molecular, biochemical, and physiological information for *H. contortus* and related nematodes (Lu et al., 2020). Meanwhile, LC−MS/MS-based proteomic analysis of excretory–secretory products of *H. contortus* showed that 878 unique proteins were identified and quantified with high confidence in key developmental stages/sexes. The proteomic analysis of glycoproteins of *H. contortus* showed that 43 proteins were identified in the adult stage, and 34 and 35 proteins were bound by Lectin galacto side-binding soluble (LGALS)-11 and LGALS-14, respectively (Sakthivel et al., 2018). Shotgun LC−MS/MS was used to identify 218 *H. contortu*s excretory and secretory proteins Haemonchus contortus Excrete Secretion Proteins (HcESPs) that interact with goat Th9 cells, which provides a new way to explore immunostimulatory antigens among HcESPs of *H. contortus* infection (Liang et al., 2022). Subsequently, iTRAQ-coupled LC−MS/MS was also applied to analyze the differentially expressed proteins (DEPs) in adult parasites between water buffalo and yellow cattle, and 131 DEPs were identified, including 46 upregulated proteins and 85 downregulated proteins (He et al., 2020). iTRAQ coupled with LC−MS/MS was also used to screen the DEPs in host cells infected with *Eimeria tenella* sporozoites, and 195 DEPs were found with high confidence, including 151 upregulated proteins and 44 downregulated proteins (Zhao et al., 2019). Furthermore, the results of comparative DIA in mice before and after *Babesia microti* infection showed that the weights of infected mice were significantly reduced, and the phosphorylation levels of proteins related to *B. microti* infection were associated with downregulated growth and development (Hu et al., 2021). Collectively, DIA deep proteomic analysis of *Toxocara canis* infection revealed that protein alterations were affected by disease development, confirming that the DIA is an ideal method for quantifying protein changes in microbiological infection (Zheng et al., 2021). A DIA quantitative proteomic approach was also used to select biomarkers in the serum for the early clinical monitoring of *B. microti* infection (Wang and Gasser, 2021). Therefore, proteomic research will provide new insights into the developmental biology of parasites, contribute to the discovery of novel regulated proteins, and enhance the understanding of pathogen−host interactions from the parasite perspective.

Although many proteomic studies have been conducted on parasites, little research has been conducted on the proteome of adult *H. contortus* specimens in domestic *Ovis ammon*. In this study, we aimed to analyze the proteomic changes and differences in adult *H. contortus* worms that developed in the abomasum of *O. ammon*. The label-free assay was used to analyze the differential proteomic profiles between adult worms in different isolates. The results showed that the identification of DEPs will offer invaluable information on the metabolic pathways and development of *H. contortus* and provide novel drug targets for the treatment of parasitic diseases.

## Materials and methods

### *H. contortus* isolates

*H. contortus* isolates were obtained from three domestic *O. ammon*. Three sick *O. ammon* died at 4–5 months of infection, and then the parasites were removed from the abomasum of the dead body. The parasite samples in this study were mainly collected from the Jinan Zoo. The fresh worms were stored in a cryogenic refrigerator and transported for proteomic analysis.

### Sample preparation

Female and male adult *H. contortus* worms were collected from three dead *O. ammon*. We analyzed the protein expression profiles of the three groups of adult worms. The groups were divided into Group 1, Group 2, and Group 3. One female adult *H. contortus* worm parasite and one male adult *H. contortus* worm parasite per group were collected and pooled to eliminate the effects of individual differences in *O. ammon*.

### Peptide preparation and high performance liquid chromatography (HPLC) fractionation

The samples were concentrated, digested, and extracted, and the protein concentration was determined by the Bradford protein assay. The supernatant from each sample, containing precisely 0.1 mg of protein, was digested with Trypsin Gold (Promega) at a 1:50 enzyme-to-substrate ratio. After 16 h of digestion at 37°C, a portion of all peptide samples was mixed equally to form the mixture sample. Then, all samples were desalted with a C18 cartridge to remove urea, and the desalted samples were dried by vacuum centrifugation. The mix sample was fractionated using a C18 column (Waters BEH C18 4.6 mm × 250 mm, 5 µm) on a Shimadzu LC 15 HPLC operating at 0.6 ml/min. Mobile phases A (2% acetonitrile, adjusted pH to 10.0 using ammonium hydroxide) and B (98% acetonitrile, adjusted pH to 10.0 using ammonium hydroxide) were used to develop a gradient elution. The eluates were monitored at UV 214 nm, collected into separate tubes every minute, and finally merged. All fractions were dried under vacuum centrifugation and reconstituted in 0.1% (v/v) formic acid (FA) in water.

### LC−MS/MS analysis

For transition library construction, shotgun proteomic analyses were performed using an ultimate 3000 Ultra High Performance Liquid Chromatography (UHPLC) system (Dionex) coupled with a Q Exactive HF mass spectrometer (Thermo Fisher) operating in the data-dependent acquisition (DDA) or DIA mode for quantification analysis. A sample volume containing 2 µg of total peptides from the fraction sample reconstituted in 0.1% FA was injected into a homemade analytical column (15 cm × 75 µm, 3 µm) in which the gradient comprised an increase from 5% to 45% solvent B [0.1% FA in 98% Aacetonitrile (ACN)] over 73 min and from 45% to 90% solvent B over 5 min and a hold at 90% solvent B for 5 min at a constant flow rate of 400 nl/min on an ultimate 3000 system. A Q Exactive HF mass spectrometer was operated in positive polarity mode with a spray voltage of 1.7 kV and capillary temperature of 320°C. Full MS scans ranging from 350 to 2,000 m/z were acquired at a resolution of 60,000 (at 200 m/z) with an automatic gain control (AGC) target value of 1 × 10^6^ and a maximum ion injection time of 30 ms. The 20 most abundant precursor ions from the full MS scan were selected for fragmentation using higher-energy collisional dissociation (HCD) fragment analysis at a resolution of 15,000 (at 200 m/z) with an AGC target value of 1 × 10^5^, a maximum ion injection time of 45 ms, a normalized collision energy of 30%, and a dynamic exclusion parameter of 20 s. The single samples were reconstituted in 0.1% FA and injected onto the ultimate 3000 UHPLC system (Dionex) coupled with an Orbitrap Q Exactive HF mass spectrometer (Thermo Fisher) operating in DIA mode. The liquid conditions were the same as above. For the DIA, MS1 resolution was set to 60,000 (at 200 m/z) and MS2 resolution was set to 15,000 (at 200 m/z). The m/z ranged from 350 to 1,200 m/z over 31 cycles. Full-scan AGC target was set to 1 × 10^6^, and injection time was set to 45 ms. The DIA settings were 27% Normalized collision energy (NCE) with a target value of 1 × 10^5^, and the maximum injection time was set to auto to allow the mass spectrometer to always operate in the parallel ion filling and detection mode.

Identification of DIA files was performed with MaxQuant 1.6.6.0 Software with default settings against the mouse UniProt reference database. The resulting list of peptides was used to create a spectral library in Skyline Software. Further analysis of DIA files was performed in Skyline Software using the default DIA protocol. The same mouse UniProt database was used to create a transition list. Quantitative analysis was also performed using the default quantification protocol. The resulting quantification data were normalized by equalizing the run medians. For quantitation, proteins were required to contain at least two quantitated unique peptides. Proteins with fold changes >1.2 or <0.83 and a significance t-test p value <0.05 between three compared groups were considered to be differentially expressed.

### Bioinformatic analysis

Then, proteins were classified by Gene Ontology (GO) annotation based on three categories: biological process, cellular component, and molecular function. The GO annotation proteome was derived from the UniProt-GOA database (http://www.ebi.ac.uk/GOA/). The Kyoto Encyclopedia of Genes and Genomes (KEGG) database was used to annotate protein pathways. We used the online KEGG automatic annotation server (KAAS) to annotate the protein’s KEGG database description and then mapped the annotation result onto the KEGG pathway database *via* KEGG mapper and KEGG online service tools. InterProScan, a sequence analysis application, was also used for protein domain annotation based on a protein sequence alignment algorithm and the InterPro domain database. A two-tailed Fisher’s exact test was employed to test the GO, KEGG pathway, and domain enrichment of the DEPs against all identified proteins. Correction for multiple hypothesis testing was carried out under standard false discovery rate control methods, and a corrected p value <0.05 was considered significant. Expression-based clustering and functional enrichment-based clustering for different protein groups were used to explore potential relationships between different protein groups with special protein functions.

## Results

### PCR amplification and sequence analysis

The PCR amplification of internal transcribed space 1 (ITS1) genes of *H. contortus* from different isolates corresponded to previous reports, with sequence lengths of approximately 1,100 bp, which is consistent with the expected fragment length. There were no nonspecific bands. Therefore, the PCR and gene sequencing results showed that the three parasite isolates were *H. contortus* isolates.

### LC−MS/MS identification of proteins

**Figure 1:**
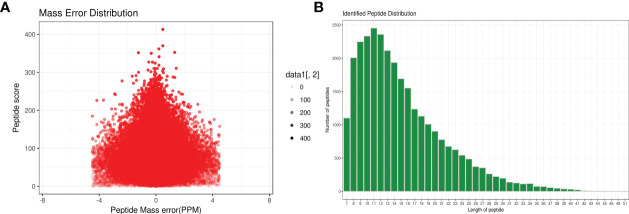
Total identified peptide distribution of *H*. *contortus*. **(A)** Mass error distribution (x axial show Parts Per Million (PPM) ; y axial show Peptide score). **(B)** Identified peptide distribution. (x axial show the length of peptides; y axial show the number peptides).

**Figure 2:**
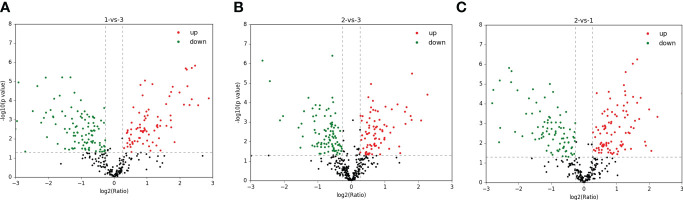
Total identified upregulated (red) and downregulated (green) protein distribution of *H. contortus*. **(A)** 1-vs-3 group; **(B)** 2-vs-3 group; **(C)** 2-vs-1 group.

A total of 1,299 adult *H. contortus* proteins were identified, and 461 proteins were quantified (**Figure 1**). All upregulated and downregulated DEPs were analyzed by pairwise comparisons (1-vs.-3, 2-vs.-3, and 2-vs.-1) (**Figure 2**). Our results showed 190, 180, and 183 DEPs, of which 82 (108), 83 (97), and 97 (86) were significantly upregulated (downregulated) DEPs, respectively. The Venn diagram shows the numbers of conserved proteins and DEPs in the different groups of *H. contortus*, and 399 proteins were shared by all three groups (**Supplementary Figure S6**).

**Figure 3:**
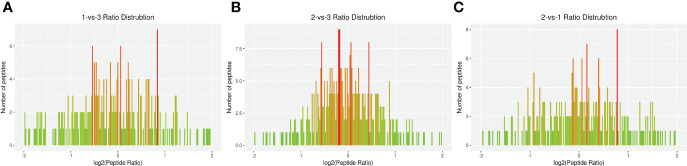
Total identified ratio distribution of *H. contortus* proteins. **(A)** 1-vs-3 Ratio Distribution; **(B)** 2-vs-3 Ratio Distribution; **(C)** 2-vs-1 Ratio Distribution. x axial show log2(Peptide Ratio), y axial show the numbers of peptides.

The DEP distribution ratios obtained from our analysis are displayed in **Figure 3**. DEPs were defined based on threshold protein quantification (1.2 and 0.05). Repeatability analysis was conducted three times in our research (**Supplementary Figure S5**).

### Functional classification of DEPs

**Figure 4:**
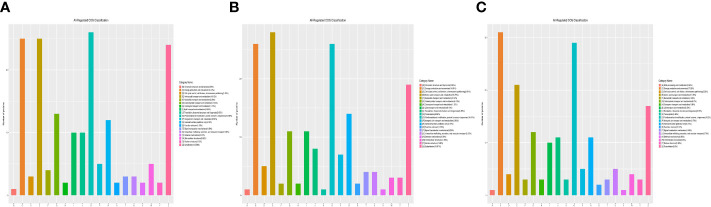
The category name of DEPs in *H*. *contortus.* Category name of panels **(A–C)** followed as different colors: (A) RNA processing modification; (B) Chromatin structure and dynamics; (C) Energy production and conversion; (D) Cell cycle control, cell division, chromosome partitioning; (E) Amino acid transport and metabolism; (F) Nucleotide transport and metabolism; (G) Carbohydrate transport and metabolism; (H) Coenzyme transport and metabolism; (I) Lipid transport and metabolism; (J) Translation, ribosomal structure, and biogenesis; (K) Transcription; (O) Posttranslational modification, protein turnover, chaperones; (P) Inorganic ion transport and metabolism; (R) General function prediction only; (S) Function unknown; (T) Signal transduction mechanisms; (U) Intracellular trafficking, secretion, and vesicular transport; (V) Defense mechanisms; (W) Extracellular structures; (Y) Nuclear structure; (Z) Cytoskeleton.

Functional classification results showed that all regulated DEPs were involved in cellular component, molecular function, biological process, and subcellular location. In the 1-vs.-3 group, 190 DEPs were mainly enriched in posttranslational modification, amino acid transport and metabolism, energy production and conversion, ribosomal structure, and biogenesis (**Figure 4A**). In addition, 180 DEPs identified in the 2-vs.-3 group were similar to those in the 1-vs.-3 group (**Figure 4B**). Finally, 183 DEPs in the 2-vs.-1 group were mainly enriched in posttranslational modification, protein turnover and chaperones, energy production and conversion, amino acid transport and metabolism, and the cytoskeleton (**Figure 4C**), which is obviously different from the enriched terms in the 1-vs.-3 group.

**Figure 5:**
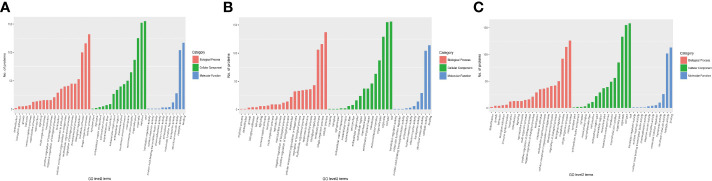
The category of DEPs including cellular components (green), molecular function (blue), and biological processes (red) in *H. contortus.* Histogram of panels **(A–C)** represents the numbers of GO classification DEPs in the three comparison groups (1-vs.-3, 2-vs.-3, and 2-vs.-1).

**Figure 6:**
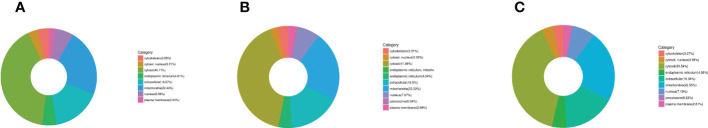
The category of DEP subcellular locations in *H. contortus*. Circle diagram of panels **(A–C)** of different color represents the category of subcellular location of DEPs, including cytoskeleton, nucleus, cytosol, endoplasmic reticulum, extracellular, mitochondria, and plasma membrane in the three comparison groups (1-vs.-3, 2-vs.-3, and 2-vs.-1).

First, three groups (1-vs.-3, 2-vs.-3, and 2-vs.-1) of DEPs were involved in more than 10 types of cellular components, mainly cell, cell part, organelle, organelle part, macromolecular complex, membrane, membrane-enclosed lumen, membrane part, extracellular region, extracellular region part, cell junction, supramolecular fiber, extracellular matrix, extracellular matrix component, synapse part, synapse, and nucleoid (**Figures 5A–C** of the green column). Second, several DEPs may play important molecular function roles in binding, catalytic activity, structural molecular activity, transporter activity, molecular function regulation, antioxidant activity, electron carrier activity, chemoattractant activity, nucleic acid binding, transcription factor activity, nutrient reservoir activity, and translation regulator activity (**Figures 5A–C** of the blue column). Third, these DEPs were involved in more than 20 types of biological processes, including cellular process, single-organism process, metabolic process, biological regulation, developmental process, regulation of biological process, multicellular organismal process, cellular component organization or biogenesis, response to stimulus, localization, negative or positive regulation of biological process, locomotion, multiorganism process, signaling, reproduction, reproductive process, immune system process, behavior, growth, biological adhesion, and detoxification (**Figures 5A–C** of the orange column). Finally, all DEPs were involved in subcellular locations, including the cytosol, endoplasmic reticulum, mitochondria, nucleus, cytoskeleton, extracellular environment, plasma membrane, cytosol, and nucleus, and the percentages of each component are displayed in **Figures 6A–C**.

### Functional enrichment of DEPs

**Figure 7:**
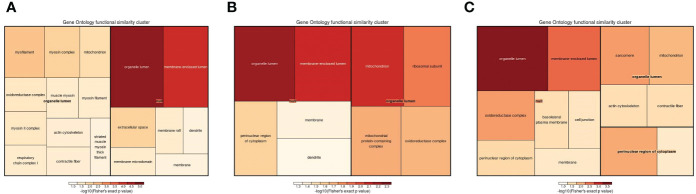
The visualization of significantly regulated GO cellular component–enriched tree map. The shade of color panels **(A–C)** representative of the number of proteins; the color range from red to blue is representative of the log10 (Fisher’s exact p value) in the three comparison groups (1-vs.-3, 2-vs.-3, and 2-vs.-1).

Cellular component, molecular function, and biological process terms and KEGG pathways were used to classify the DEPs by GO enrichment analysis. Initially, cellular component enrichment results revealed 43 (69) significantly upregulated (downregulated) DEPs among 704 proteins in the 1-vs.-3 group (**Figure 7A**). The top 5 enriched cellular components were organelle lumen, membrane-enclosed lumen, intracellular organelle lumen, cytoskeleton, and cytoskeletal part, with more than 25 DEPs (**Figure 7A**). Then, we analyzed the cellular component enrichment in the 2-vs.-3 group, which had 50 (54) significantly upregulated (downregulated) DEPs (**Figure 7B**). The top 5 enriched cellular components were mitochondrion, organelle lumen, membrane-enclosed lumen, intracellular organelle part, and mitochondrial matrix, and the number of DEPs associated with each term was more than 30 (**Figure 7B**). In the end, we found 58 (44) significantly upregulated (downregulated) DEPs in the 2-vs.-1 group, in which the number of upregulated DEPs exceeded the number of downregulated DEPs (**Figure 7C**). The top 5 enriched cellular components were organelle part, organelle lumen, membrane-enclosed lumen, intracellular organelle lumen, and cytoskeleton, and the number of DEPs associated with each term was more than 40 (**Figure 7C**).

**Figure 8:**
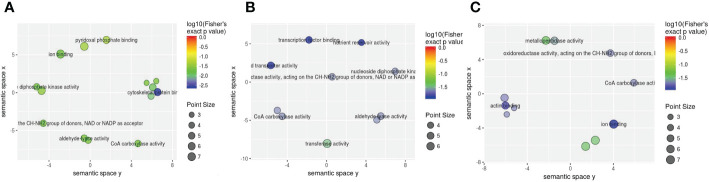
The visualization of significantly regulated GO molecular function–enriched scatter. The point size of panels **(A–C)** is representative of the number of proteins; the color range from red to blue is representative of the log10 (Fisher’s exact p value) in the three comparison groups (1-vs.-3, 2-vs.-3, and 2-vs.-1).

Subsequently, molecular function enrichment results revealed 105 DEPs among 700 proteins in the three groups. First, we determined all enriched GO molecular function terms for the DEPs in the 1-vs.-3 group, including 45 (60) significantly upregulated (downregulated) DEPs (**Figure 8A**). The top 2 enriched molecular functions were ion binding and anion binding, with more than 50 DEPs (**Figure 8A**). Compared with the 1-vs.-3 group, 54 (53) significantly upregulated (downregulated) DEPs were found in the 2-vs.-3 group (**Figure 8B**). The top 2 enriched molecular functions included transferase activity and cofactor binding, with more than 20 DEPs associated with each group (**Figure 8B**). Finally, the molecular function enrichment results showed 58 (43) significantly upregulated (downregulated) DEPs in the 2-vs.-1 group (**Figure 8C**). The top 5 enriched molecular functions were ion binding, cation binding, adenyl ribonucleotide binding, adenyl nucleotide binding, and ATP binding, with more than 40 DEPs associated with each (**Figure 8C**).

**Figure 9:**
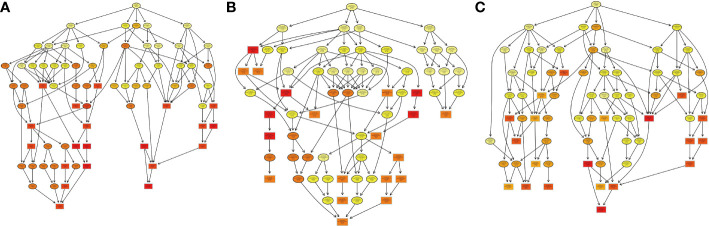
The visualization of significant biological processes–enriched GO terms. Each cycle content included GO number of proteins and subcellular locations in *H. contortus*.

GO enrichment analysis was also used to determine the associations of 624 DEPs in the three groups with biological processes. The results showed that 82, 86, and 80 DEPs, of which 34 (48), 42 (44), and 44 (36) were significantly upregulated (downregulated) DEPs, were analyzed among the pairwise comparisons (1-vs.-3, 2-vs.-3, and 2-vs.-1 group), respectively (**Figures 9A–C**). The top 5 enriched biological processes in the 1-vs.-3 group were nucleotide, nucleotide phosphate, ribonucleotide, purine-containing compound, and purine ribonucleotide metabolic processes (**Figure 9A**), while the top 5 enriched biological processes in the 2-vs.-3 group were single-organism, metabolic process, oxoacid, organic, and carboxylic metabolic processes (**Figure 9B**). However, in the 2-vs.-1 group, the top 2 enriched biological processes were oxoacid metabolic process and single-organism catabolic process, with more than 25 DEPs associated with each group (**Figure 9C**).

**Figure 10:**
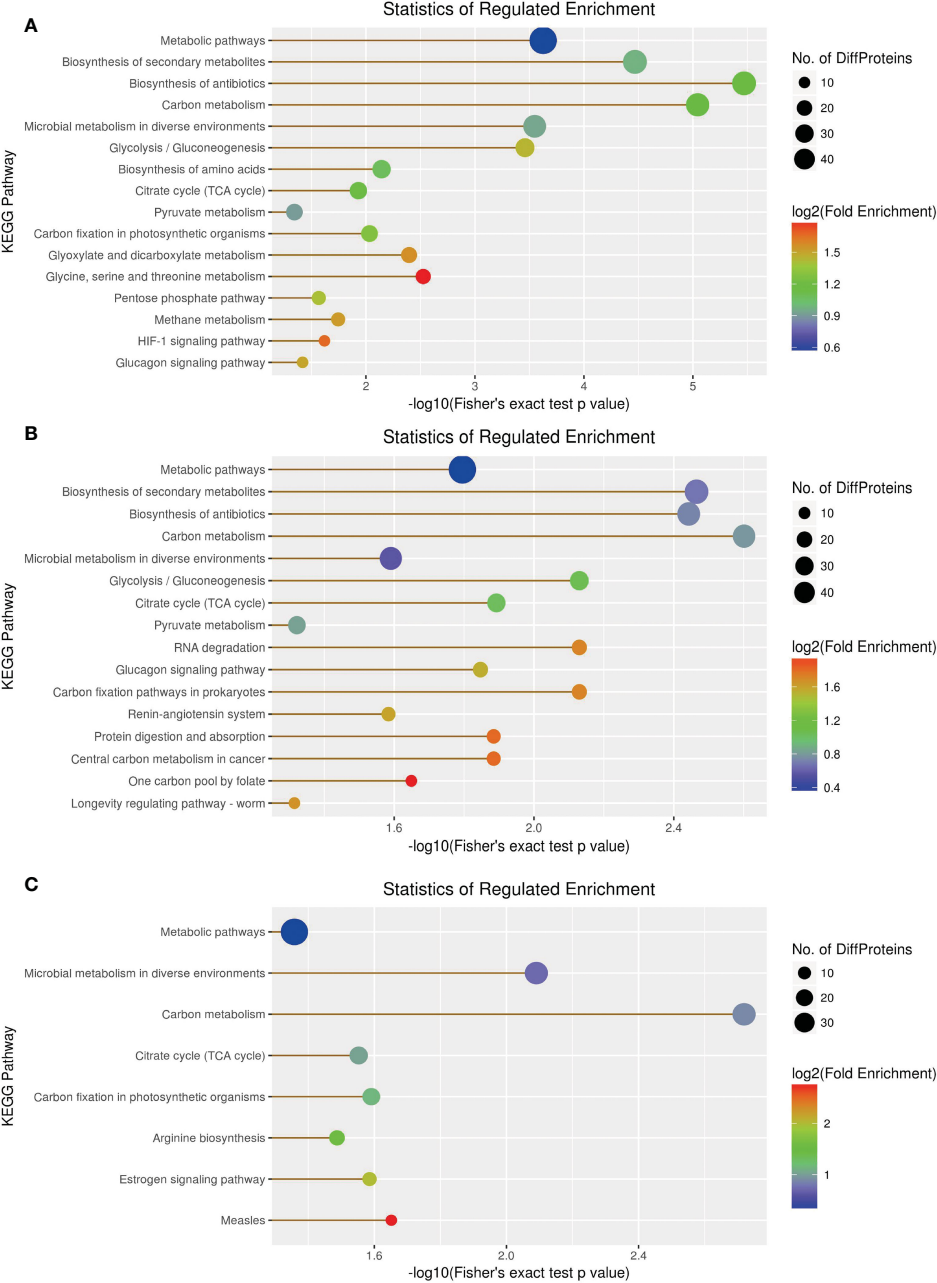
The visualization of significantly KEGG-enriched GO terms. **(A)** 1-vs-3 Ratio Distribution; **(B**) 2-vs-3 Ratio Distribution; **(C)** 2-vs-1 Ratio Distribution. x axial show -log10(Fisher's exact test p value), y axial show the KEGG Pathway.

KEGG enrichment analysis revealed 66 DEPs, including 23 (43) significantly upregulated (downregulated) DEPs among 419 proteins in the 1-vs.-3 group (**Supplementary Tables 1**–**3**). The top 5 enriched pathways were metabolic pathways, biosynthesis of secondary metabolites, biosynthesis of antibiotics, carbon metabolism, and microbial metabolism in diverse environments (**Figure 10A**). In contrast, KEGG enrichment analysis of the 2-vs.-3 group revealed 79 DEPs, including 35 (44) significantly upregulated (downregulated) DEPs **Supplementary Tables 1, 2**). The top 5 enriched pathways in the 2-vs.-3 group were the same as those in the 1-vs.-3 group (**Figure 10B**). KEGG enrichment analysis in the 2-vs.-1 group showed 63 DEPs, including 39 (34) significantly upregulated (downregulated) DEPs among 419 proteins (**Supplementary Tables 1**–**3**) (**Figure 10C**). In comparison with the previous two groups, the top 5 enriched pathways were metabolic pathways, microbial metabolism in diverse environments, carbon metabolism, citrate cycle [tricarboxylic acid cycle (TCA cycle) cycle], and carbon fixation (**Figure 10C**).

**Figure 11:**
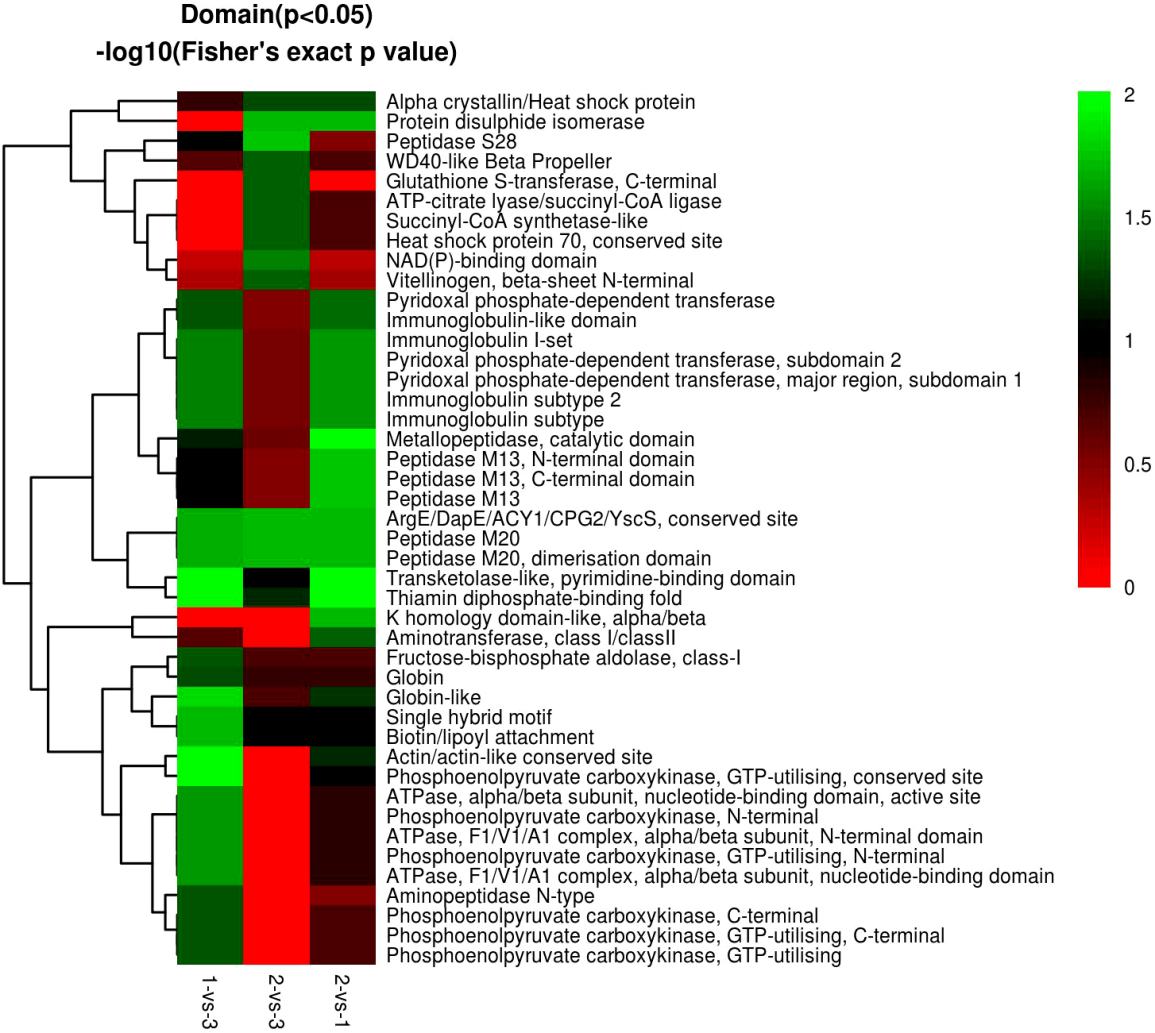
The visualization of significantly domain-enriched cluster. From red to green (score 0-2), it means the Domain (P<0.05) -log10(Fisher's exact p value).

In the domain enrichment analysis, we first calculated 177 DEPs among 1,196 quantified proteins, including 76 (101) upregulated (downregulated) DEPs. We found several important functional domains in the 1-vs.-3 group, including pyridoxal phosphate-dependent transferase subdomain 2, pyridoxal phosphate-dependent transferase major region, immunoglobulin subtype 2, immunoglobulin subtype, and immunoglobulin I-set (**Figure 11**). Then, we analyzed the domain enrichment in the 2-vs.-3 group, with 171 DEPs, including 82 (89) upregulated (downregulated) DEPs. The results showed that the number of NAD(P)-binding domains was greater than that of other domains (**Figure 11**). Finally, we calculated 170 DEPs, with 93 (77) upregulated (downregulated) DEPs, in the 2-vs.-1 group. We also found several important functional domains, including pyridoxal phosphate-dependent transferase subdomain 2, pyridoxal phosphate-dependent transferase major region, pyridoxal phosphate-dependent transferase, immunoglobulin-like domain, immunoglobulin subtype 2, immunoglobulin subtype, immunoglobulin I-set, and alpha crystalline/heat shock protein, which are similar to the domains identified in the 1-vs.-3 group (**Figure 11**).

## Discussion

Haemonchosis is a widespread epidemic in developed and developing countries, including the United Kingdom, New Zealand, United States, Denmark, Turkey, Australia, and other countries (Kaminsky, 2003; Muchiut et al., 2018). According to reports from 2010 to 2013, the *H. contortus* infection rate was 60% in semiarid regions of India (Besier et al., 2016b). In 2016, an investigation of haemonchosis in Turkey demonstrated that the infection rate was 24.8% (Akkari et al., 2013). The distribution of haemonchosis has also been recorded in China, which is a large civilized country with a long history of 5,000 years. The comfortable temperature and humidity in China make it very suitable for the development of cattle, sheep, and other livestock breeding industries. Meanwhile, a large amount of animal farming also provides a suitable environment for the development and transmission of *H. contortus*, and *H. contortus* infection has been reported in more than 20 provinces and regions, including Inner Mongolia, Hunan, Guangxi, Shanxi, Shaanxi, Anhui, Qinghai, Beijing, Xinjiang, and Jiangsu provinces, with infection rates of 17.4%–100% (Yin et al., 2013; Shen et al., 2019). Although a large number of studies have reported haemonchosis in sheep, cattle, and goats, few studies have focused on wild animals, especially *O. ammon*, in animal zoos.

Previous reports have shown that the development of proteomics differs significantly in different hosts and isolates (Monteiro et al., 2017; Camargo et al., 2018; Di Maggio et al., 2022; Tasbihi et al., 2020). Although there have been several reports on *H. contortus* infection in sheep, goats, and other ruminant animals, few studies have reported on DEPs of *H. contortus* worms in domestic *O. ammon*. In the present study, we used the label-free proteomic method to identify DEPs in three isolates of *H. contortus* at the protein level. A total of 1,299 adult *H. contortus* proteins were identified, and 461 proteins were quantified with confidence level (**Figure 2**), of which 82 (108), 83 (97), and 97 (86) were significantly upregulated (downregulated) proteins in pairwise comparisons (1-vs.-3, 2-vs.-3, and 2-vs.-1, respectively). The number of identified adult proteins was 847, 910, and 1,016 in the three groups, respectively. To understand how the mechanism of parasite and host interaction occurs at the protein level, all significantly upregulated and downregulated DEPs of *H. contortus* are displayed in **Supplementary Tables 1**–**3**.

**Figure 12:**
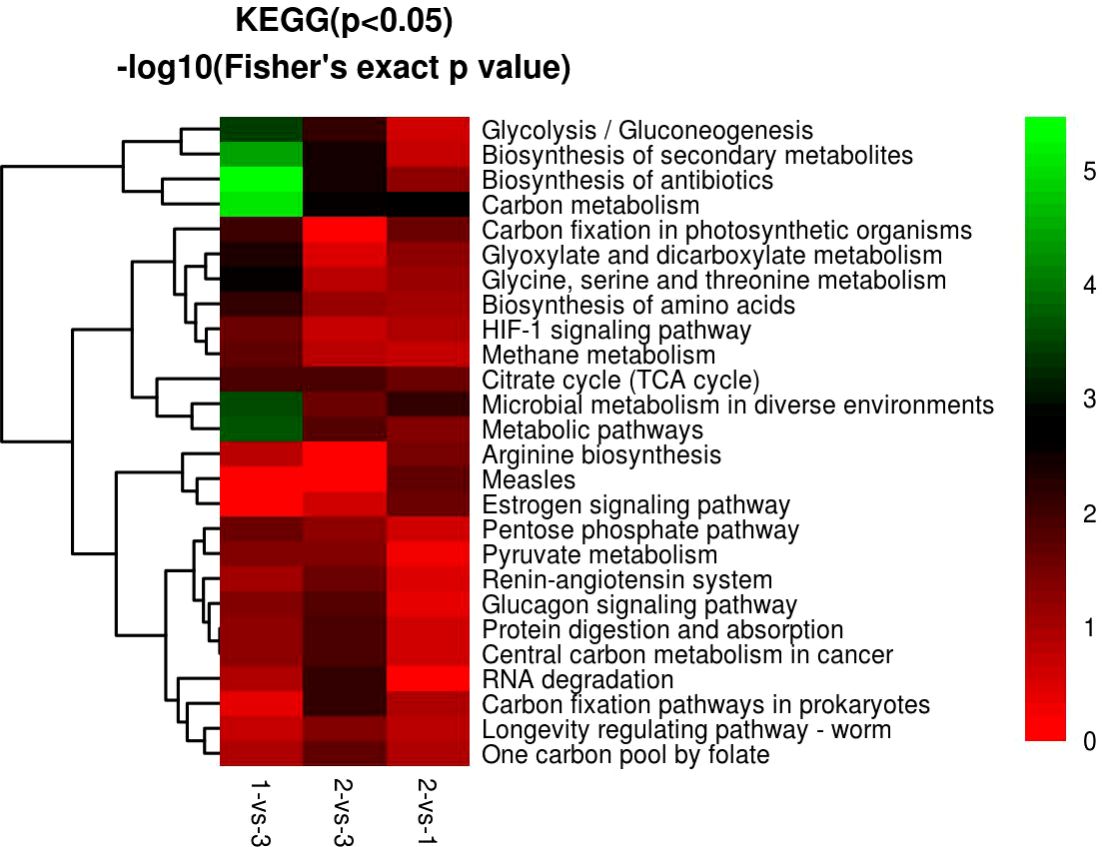
GO KEGG function cluster of 1-vs.-3, 2-vs.-3, and 2-vs.-1. From red to green (score 0-5), it means the KEGG (P<0.05) -log10(Fisher's exact p value).

KEGG enrichment analysis showed that most of the DEPs focused on carbon metabolism, biosynthesis of antibiotics, biosynthesis of secondary metabolites, and metabolic pathways in *H. contortus* (**Figures 10**, **12**). Serine hydroxymethyl transferase (SHMT), dihydrolipoyl dehydrogenase (DLD), and transket pyr domain-containing protein (TKPD) simultaneously played important roles in carbon metabolism in the three groups, and these proteins were involved in metabolism, with downregulated functions in the 1-vs.-3 and 2-vs.-3 groups and upregulated functions in the 2-vs.-1 group. SHMT is a pyridoxal phosphate (PLP)-dependent enzyme that catalyzes the reversible conversion of serine and tetrahydrofolate to glycine and methylenetetrahydrofolate (Mukherjee et al., 2006). It was initially reported in an amitochondriate of *Trichomonas vaginalis* with a deep-branching unicellular protist. The identification and biochemical characterization showed that the SHMT protein possesses a putative N-terminal hydrogenosomal presequence and localizes to hydrogenosomes (Mukherjee et al., 2006). High-grade serous (HGS) ovarian cancer research showed that SHMT1 can stimulate pro-oncogenic cytokine expression through sialic acid to promote tumor growth and progression (Gupta et al., 2017). Compared with SHMT1, SHMT2 also exists in various cancers *via* the conversion of serine and glycine in mitochondria to support cancer cell proliferation. A previous report showed that deacetylation of SHMT2 by sirtuin (SIRT3) promotes colorectal carcinogenesis (Wei et al., 2018). Except for tumor cells, mutant eggs in *Drosophila* showed SHMT-dependent metabolites in amounts that suffice for early development during rapid syncytial cell cycles (Winkler et al., 2017). DLD is an old flavin-dependent enzyme that is critical for energy metabolism and redox balance, and an early report identified a mutant deficient in DLD in *Escherichia coli* and studied its characteristics (Alwine et al., 1973). Related research identified a DLD-binding protein as a component of the pyruvate dehydrogenase complex of the anaerobic parasitic nematode *Ascaris suum* (Klingbeil et al., 1996). In addition, DLD can be involved in pathogen−host interactions with the invasion of *Paracoccidioides brasiliensis* and *Paracoccidioides lutzii* accompanied by paracoccidioidomycosis (Landgraf et al., 2017). In contrast to the two proteins above, there has been little research on TKPD in the PubMed database until now. Therefore, TKPD is also a mystery in *H. contortus* infection and may be involved in transport function in the intracellular or extracellular environment.

Both isocitrate dehydrogenase (IDH) nicotinamide adenine dinucleotide phosphate (NADP) and pyruvate dehydrogenase E1 component subunit beta (PHE1B) were commonly significantly upregulated and downregulated in metabolic pathways in *H. contortus*. Previous research demonstrated that NADP-dependent IDH of *Plasmodium falciparum* (pfICDH) generates nicotinamide adenine dinucleotide phosphate H (NADPH) and is crucial for parasite survival and pathogenicity, which is also an important drug target in *P. falciparum* infection (Chan and Sim, 2003). The biochemical and structural features of leishmanial IDHs showed that they are potential new therapeutic targets of metabolic processes in *Leishmania* parasites (Giordana and Nowicki, 2020). Label-free LC−MS/MS analysis of DEPs between *Trichinella spiralis* muscle larvae (ML) and intestinal infective larvae (IIL) showed that NADP-dependent IDH might be a molting-related gene, which will be helpful for developing vaccines and new drugs against *Trichinella spiralis (T. spiralis)* (Ren et al., 2019). Compared with IDH (NADP), pyruvate dehydrogenase is involved in regulating energy metabolism, growth, differentiation, and drug targets and interacts with effectors in intracellular and extracellular environments (Lander et al., 2018; Yang et al., 2019; Ferrarini et al., 2021; Chan et al., 2022). Until now, there has been little research on PHE1B in parasites and microbiology.

Other groups of DEPs were involved in the biosynthesis of antibiotics in *H. contortus*, including SHMT, DLD, TKPD, and PHE1B. SHMT not only plays a significant role in carbon metabolism but also is involved in the biosynthesis of antibiotics, which could be extensively applied in the treatment of cancers and the development of antibiotics (Wang et al., 2022). Comparative transcriptome analysis of *Streptomyces nodosus* mutants showed that upregulated pyruvate dehydrogenase can accelerate the consumption of glucose and has great effects on the accumulation of precursors, in which antibiotics are derived from microbial secondary metabolites (Huang et al., 2021). A previous report showed that intermediary metabolism and respiration by DLD were affected by ethambutol and could be explored as additional drug targets (Ghiraldi-Lopes et al., 2019). The function of TKPD is also unknown, and further research is needed in the future.

Although our study identified several novel proteins in *H. contortus*, there are still several specific features and limitations of our research that should be noted. In the present research, we adopted three adult isolates but not the complete life history stage of *H. contortus* because we did not acquire the different stages of the parasite. On the other hand, we used three novel and natural *H. contortus* infection isolates instead of experimental isolates to compare the different proteomes; however, we did not determine the standard strain involved in our research.

In the present study, we used the label-free proteomic method combined with LC−MS/MS to identify DEPs in different *H. contortus* isolates from *O. ammon*. First, the bioinformatic analysis showed that the DEPs differed significantly among the three individual isolates, and some DEPs may play important roles in carbon metabolism, biosynthesis of antibiotics, and metabolic pathways. Furthermore, other DEPs were also involved in the biosynthesis of secondary metabolites, protein digestion and absorption, glyoxylate and dicarboxylate metabolism, microbial metabolism in diverse environments, and methane metabolism. Finally, we acquired a large number of novel proteins by proteomic analysis in *H. contortus*, which could serve as new drug targets and vaccine components in the future. These new drug targets and vaccine components may be helpful to control *H. contortus* infection in susceptible animals.

In summary, comparative proteomic analysis of adult worms displayed significant differences in three different individual isolates, which helps to improve our understanding of the growth and metabolic mechanisms of *H. contortus* in different individuals and relative natural environments and provides novel drug targets for the treatment of parasitic diseases.

## Data availability statement

The datasets presented in this study can be found in online repositories. The names of the repository/repositories and accession number(s) can be found below: The mass spectrometry proteomics data of my article (1087210) have been deposited to the ProteomeXchange Consortium (http://proteomecentral.proteomexchange.org) *via* the iProX partner repository with the dataset identifier ID: PXD038346.

## Ethics statement

The parasites were removed from three dead mouflons. All protocols related to animals were carried out based on the guidelines of the Association for Assessment and Accreditation of Laboratory Animal Care International.

## Author contributions

GL designed the study and drafted the manuscript. QL, ZH, PW and YL collected the animal specimens and supported the experiment. All persons who have made substantial contributions to the work are reported in the manuscript.
