# Supplementary material for: Comparative proteomics analysis of adult Haemonchus contortus isolates from Ovis ammon
Source: Front Cell Infect Microbiol. 2023 Mar 16;13:1087210. doi: 10.3389/fcimb.2023.1087210 (PMC10061303; doi:10.3389/fcimb.2023.1087210)
Supplement: Supplementary file 1 [file DataSheet_1.docx]

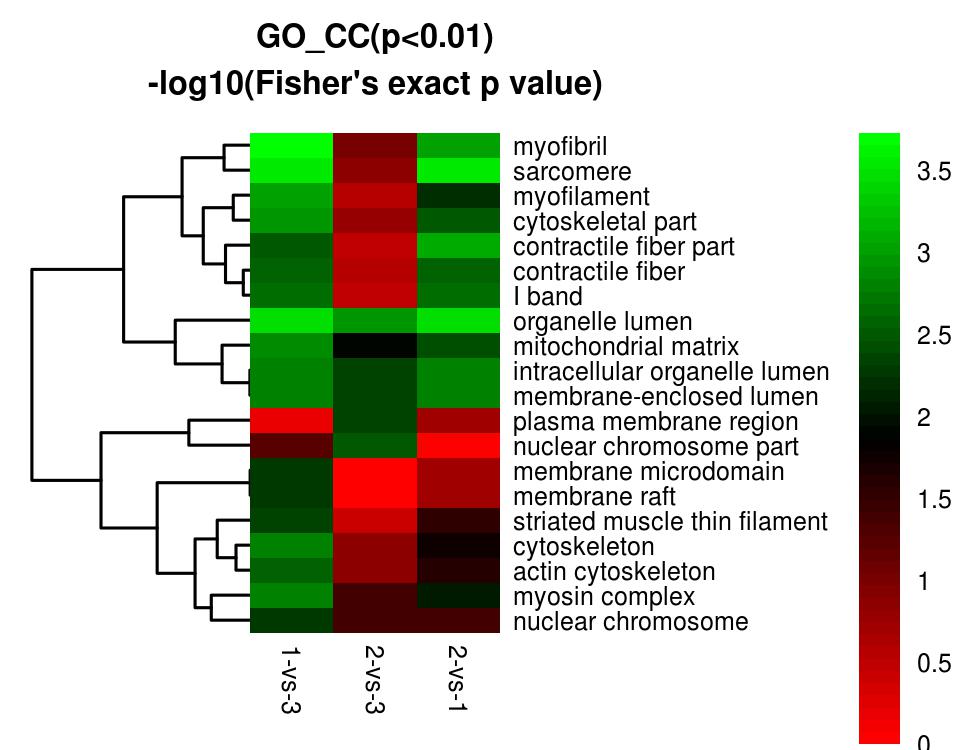


Supplementary Figure 1. GO Cellular component function cluster of three groups.


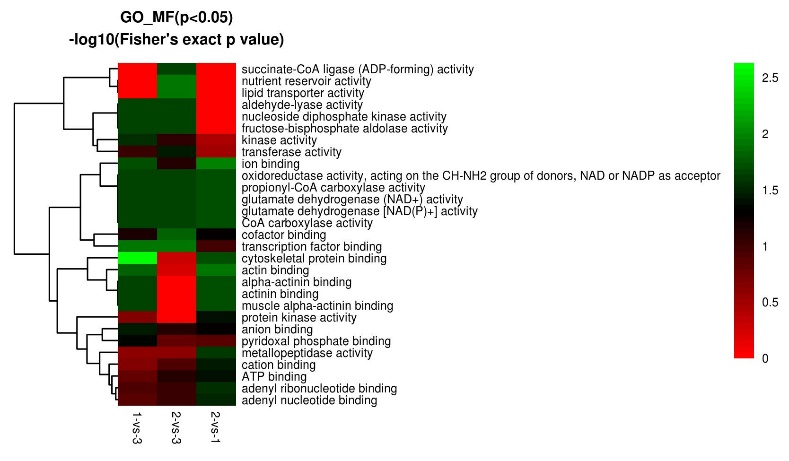


Supplementary Figure 2. GO Molecular function cluster of three groups.


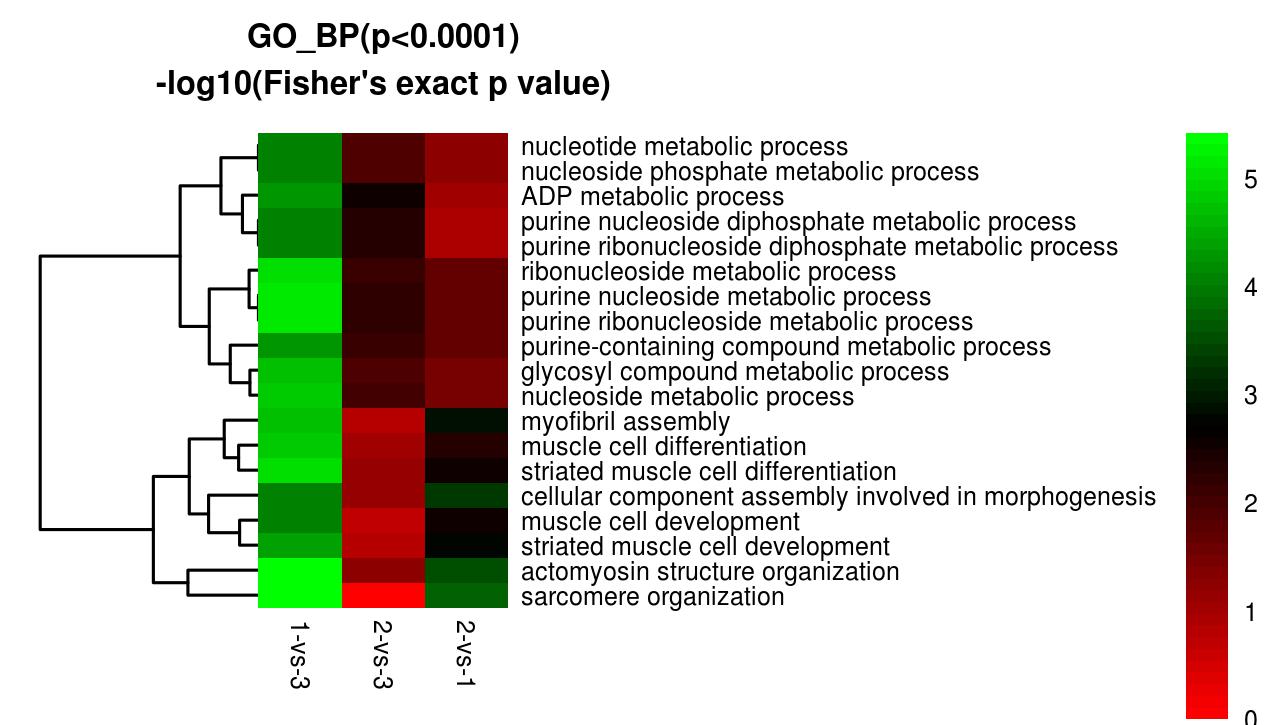


Supplementary Figure 3. GO Biological process function cluster of three groups.


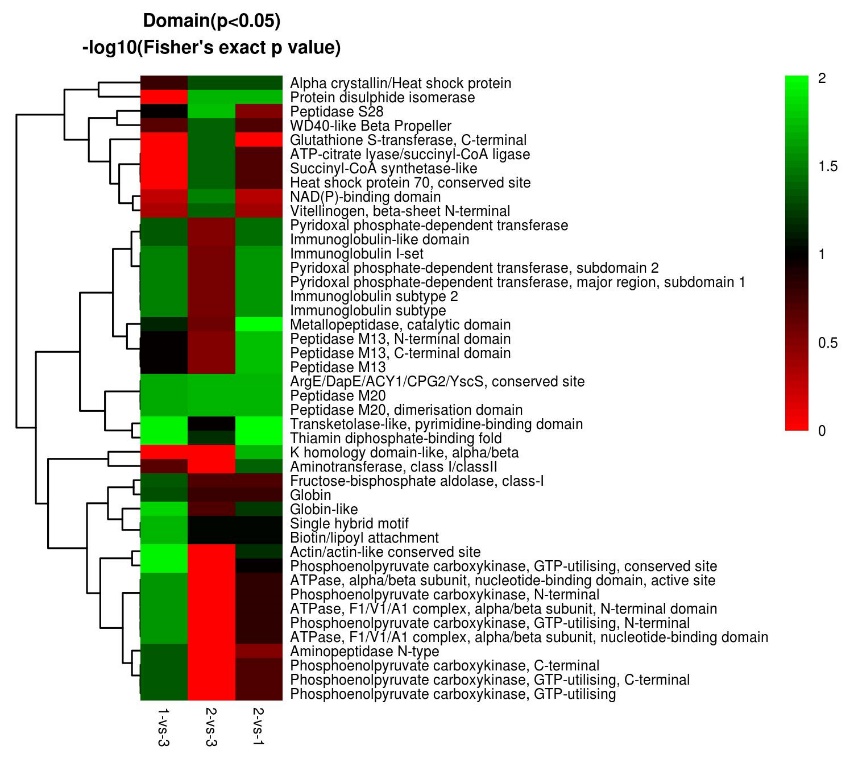


Supplementary Figure 4. GO Domain function cluster of three groups.


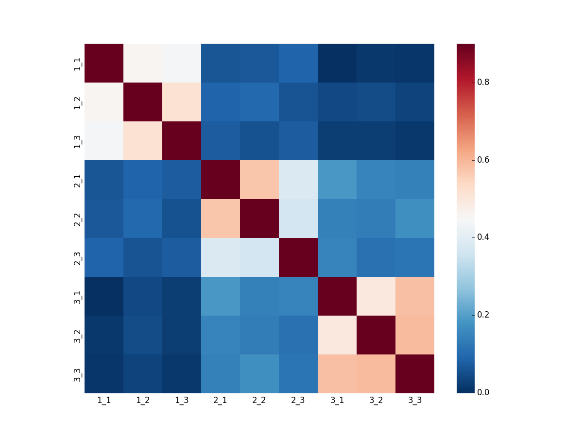


Supplementary Figure 5. Repeatability analysis of 1 vs 3, 2 vs 3 and 2 vs 1 groups.


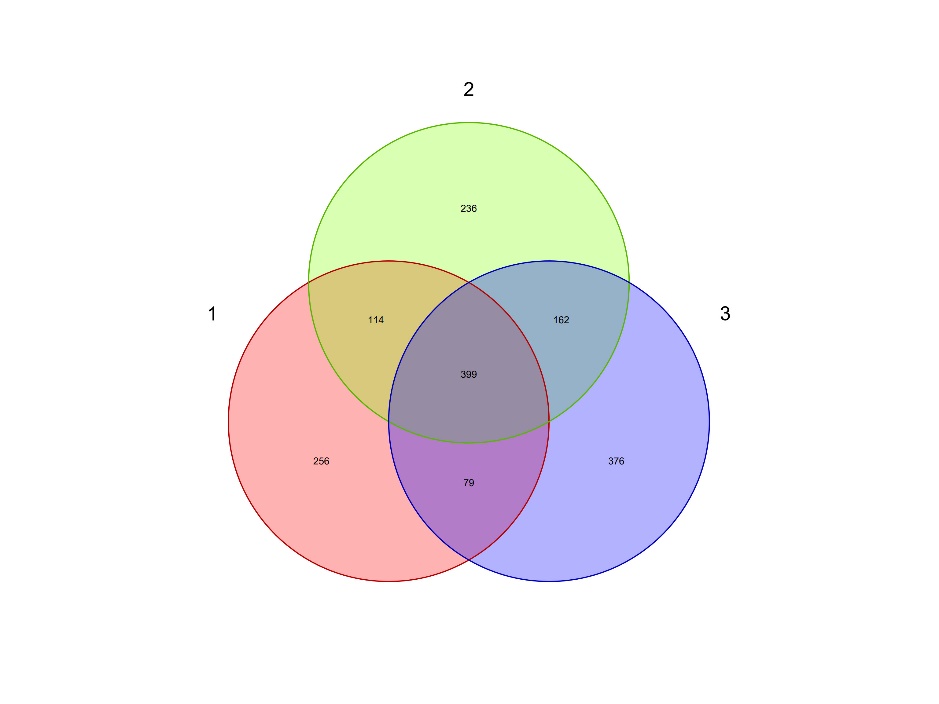
 Supplementary Figure 6. Venn diagram showing the numbers of conserved and DEPs by group1, group2 and group3 in H.contortus. (The results showed that 399 proteins were shared in three groups)
