# Supplementary material for: Comparative proteomics analysis of adult Haemonchus contortus isolates from Ovis ammon
Source: Front Cell Infect Microbiol. 2023 Mar 16;13:1087210. doi: 10.3389/fcimb.2023.1087210 (PMC10061303; doi:10.3389/fcimb.2023.1087210)
Supplement: Supplementary file 2 [file Table_1.docx]

Supplementary Table1. All-regulated DEPs of [*Haemonchus contortus*](https://www.uniprot.org/taxonomy/6289) in 1-vs-3 group

| Category_  Name | Description OS=  [*Haemonchus contortus*](https://www.uniprot.org/taxonomy/6289) | ProteinIDs | Regulated-Stage | Fisher's exact test p value |
| --- | --- | --- | --- | --- |
| Biosynthesis of secondary metabolites | Pyruvate dehydrogenase E1 component subunit alpha;  Fructose-bisphosphate aldolase;  Aminomethyltransferase;  Alpha-1,4 glucan phosphorylase ;  Aspartate aminotransferase;  Glycine cleavage system H protein;  Malate dehydrogenase;  Aldo keto reductase domain containing protein;  Ornithine aminotransferase | A0A7I4Y152;  R4H2V1;  A0A7I4YTF8;  A0A0N4WWC4;  A0A7I4YXS0;  A0A7I4XVR6;  A0A6F7P888;  A0A7I4Y0C3;  A0A6F7P322 | Up | 0.030 |
| Biosynthesis of antibiotics | Aminomethyltransferase;  Pyruvate dehydrogenase E1 component subunit alpha;  Glycine cleavage system H protein;  Malate dehydrogenase;  Aldo keto reductase domain containing protein;  Aspartate aminotransferase;  Fructose-bisphosphate aldolase ;  Ornithine aminotransferase | A0A7I4YTF8;  A0A7I4Y152;  A0A7I4XVR6;  A0A6F7P888;  A0A7I4Y0C3;  A0A7I4YXS0;  R4H2V1;  A0A6F7P322 | Up | 0.033 |
| Protein digestion and absorption | Intestinal prolyl carboxypeptidase 1；  Peptidase S28 domain containing protein | A5CG76;  W6NFT9 | Up | 0.038 |
| Glyoxylate and dicarboxylate metabolism | Glycine cleavage system H protein；  Malate dehydrogenase；  Aminomethyltransferase | A0A7I4XVR6;  A0A6F7P888;  A0A7I4YTF8 | Up | 0.043 |
| Metabolic pathways | Pyruvate dehydrogenase E1 component subunit alpha；  Fructose-bisphosphate aldolase；  CYTOSOL_AP domain-containing protein；  Aminomethyltransferase；  Alpha-1,4 glucan phosphorylase；  NADH dehydrogenase 1 alpha subcomplex subunit 5；Hydroxyacyl-coenzyme A dehydrogenase, mitochondrial；  Aspartate aminotransferase；  Adenine phosphoribosyl transferase；  Glycine cleavage system H protein；  Adenylate kinase isoenzyme 1；  Malate dehydrogenase；  Aldo keto reductase domain containing protein；  Ornithine aminotransferase | A0A7I4Y152;  R4H2V1;  A0A7I4YUK0;  A0A7I4YTF8;  A0A0N4WWC4;  A0A7I4YQ96;  A0A7I4Z0Q7;  A0A7I4YXS0;  A0A7I4XU04;  A0A7I4XVR6;  A0A7I4Z188;  A0A6F7P888;  A0A7I4Y0C3;  A0A6F7P322 | Up | 0.046 |
| Carbon metabolism | Succinate dehydrogenase flavoprotein subunit, mitochondrial;  Transket_pyr domain-containing protein;  Serine hydroxymethyl transferase;  Dihydrolipoyl dehydrogenase;  Fumarate hydratase;  Glutamate dehydrogenase;  Acetyltransferase component of pyruvate dehydrogenase complex;  Threonine ammonia-lyase;  Isocitrate dehydrogenase [NADP];  Phosphopyruvate hydratase;  Glycine cleavage system P protein;  Malic enzyme;  Pyruvate kinase;  Pyruvate kinase;  Glucose-6-phosphate isomerase;  Oxoglutarate dehydrogenase (succinyl-transferring);  Fructose-bisphosphate aldolase;  Fructose-bisphosphatase;  Phosphoglycerate kinase | A0A7I4YZY5;  A0A7I4YJF;  A0A0N4W4W6;  A0A7I4YM01;  A0A6F7PVQ8;  A0A6F7PEZ2;  A0A7I4YI94;  A0A7I5EBZ2;  A0A7I5E865;  A0A7I4Z2S6;  U6PW36;  A0A7I4Y9K3;  A0A7I4Y809;  A0A7I4YQ11;  A0A7I4XWS3;  A0A7I4YHD0;  A0A7I4Y9W7;  A0A7I4Z5T7;  A0A7I4Z1I0 | Down | 3.54E-06 |
| Biosynthesis of antibiotics | Succinate dehydrogenase flavoprotein subunit, mitochondrial;  Serine hydroxymethyl transferase;  Dihydrolipoyl dehydrogenase;  Pyruvate dehydrogenase E1 component subunit beta;  Fructose-bisphosphate aldolase;  Fumarate hydratase;  Acetyltransferase component of pyruvate dehydrogenase complex;  Threonine ammonia-lyase;  Isocitrate dehydrogenase [NADP];  Phosphoglycerate kinase;  Phosphopyruvate hydratase;  Glycine cleavage system P protein;  Pyruvate kinase;  Glucose-6-phosphate isomerase;  Nucleoside diphosphate kinase;  Transket_pyr domain-containing protein;  Oxoglutarate dehydrogenase (succinyl-transferring);  Fructose-bisphosphatase | A0A7I4YZY5;  A0A0N4W4W6;  A0A7I4YM01;  A0A6F7PVQ8;  A0A7I4Y9W7;  A0A6F7PEZ2;  A0A7I5EBZ2;  A0A7I5E865;  A0A7I4Z2S6;  A0A7I4Z1I0;  U6PW36;  A0A7I4Y9K3;  A0A7I4YQ11;  A0A7I4XWS3;  A0A7I4XSS8;  A0A7I4YJF1;  A0A7I4YHD0;  A0A7I4Z5T7 | Down | 6.82E-05 |
| Microbial metabolism in diverse environments | Succinate dehydrogenase flavoprotein subunit, mitochondrial;  Serine hydroxymethyl transferase;  Dihydrolipoyl dehydrogenase;  Glutamine synthetase；  Pyruvate dehydrogenase E1 component subunit beta ；  Fumarate hydratase；  Acetyltransferase component of pyruvate dehydrogenase complex；  Isocitrate dehydrogenase [NADP]；  Phosphopyruvate hydratase；  Malic enzyme；  Pyruvate kinase；  Transket_pyr domain-containing protein；  Glucose-6-phosphate isomerase；  Oxoglutarate dehydrogenase (succinyl-transferring)；  Fructose-bisphosphate aldolase；  Fructose-bisphosphatase；  Phosphoglycerate kinase | A0A7I4YZY5;  A0A0N4W4W6;  A0A7I4YM01;  A0A7I4YU77;  A0A6F7PVQ8;  A0A6F7PEZ2;  A0A7I5EBZ2;  A0A7I4Z2S6;  U6PW36;  A0A7I4Y809;  A0A7I4YQ11;  A0A7I4YJF1;  A0A7I4XWS3;  A0A7I4YHD0;  A0A7I4Y9W7;  A0A7I4Z5T7;  A0A7I4Z1I0 | Down | 0.0004 |
| Biosynthesis of secondary metabolites | Fumarate hydratase;  Succinate dehydrogenase flavoprotein subunit, mitochondrial;  Serine hydroxymethyl transferase;  Dihydrolipoyl dehydrogenase；  Acetyltransferase component of pyruvate dehydrogenase complex;  Threonine ammonia-lyase;  Nucleoside diphosphate kinase;  Pyruvate dehydrogenase E1 component subunit beta;  Isocitrate dehydrogenase [NADP];  Phosphoglycerate kinase;  Phosphopyruvate hydratase;  Glycine cleavage system P protein;  Pyruvate kinase;  Glucose-6-phosphate isomerase;  Fructose-bisphosphate aldolase;  Fructose-bisphosphatase;  Transket_pyr domain-containing protein;  Oxoglutarate dehydrogenase (succinyl-transferring) | A0A6F7PEZ2;  A0A7I4YZY5;  A0A0N4W4W6;  A0A7I4YM01;  A0A7I5EBZ2;  A0A7I5E865;  A0A7I4XSS8;  A0A6F7PVQ8;  A0A7I4Z2S6;  A0A7I4Z1I0;  U6PW36;  A0A7I4Y9K3;  A0A7I4YQ11;  A0A7I4XWS3;  A0A7I4Y9W7;  A0A7I4Z5T7;  A0A7I4YJF1;  A0A7I4YHD0 | Down | 0.0007 |
| Glycolysis / Gluconeogenesis | Pyruvate kinase;  Dihydrolipoyl dehydrogenase;  Pyruvate dehydrogenase E1 component subunit beta;  Acetyltransferase component of pyruvate dehydrogenase complex; Phosphopyruvate hydratase;  Glucose-6-phosphate isomerase;  Phosphoglycerate kinase;  Fructose-bisphosphate aldolase;  Fructose-bisphosphatase | A0A7I4YQ11;  A0A7I4YM01;  A0A6F7PVQ8;  A0A7I5EBZ2;  U6PW36;  A0A7I4XWS3;  A0A7I4Z1I0;  A0A7I4Y9W7;  A0A7I4Z5T7 | Down | 0.0008 |
| Metabolic pathways | Fumarate hydratase;  Glycine cleavage system P protein;  Aminotran_1_2 domain-containing protein;  ATP synthase subunit alpha;  Serine hydroxymethyl transferase;  Dihydrolipoyl dehydrogenase;  Acetyltransferase component of pyruvate dehydrogenase complex; Threonine ammonia-lyase;  Glutamine synthetase;  Propionyl-CoA carboxylase alpha chain, mitochondrial;  Nucleoside diphosphate kinase;  Succinate dehydrogenase flavoprotein subunit, mitochondrial; Adenosyl homocysteinase;  Pyruvate dehydrogenase E1 component subunit beta;  Isocitrate dehydrogenase [NADP];  Phosphopyruvate hydratase;  Malic enzyme;  Pyruvate kinase;  Glucose-6-phosphate isomerase;  H(+)-transporting two-sector ATPase;  Fructose-bisphosphate aldolase;  Fructose-bisphosphatase;  Transket_pyr domain-containing protein;  Glutamate dehydrogenase;  ATP synthase subunit beta;  Phosphoglycerate kinase;  Oxoglutarate dehydrogenase (succinyl-transferring) | A0A6F7PEZ2;  A0A7I4Y9K3;  A0A7I4YNV2;  A0A7I4Y004;  A0A0N4W4W6;  A0A7I4YM01;  A0A7I5EBZ2;  A0A7I5E865;  A0A7I4YU77;  A0A7I5E751;  A0A7I4XSS8;  A0A7I4YZY5;  A0A7I4XY18;  A0A6F7PVQ8;  A0A7I4Z2S6;  U6PW36;  A0A7I4Y809;  A0A7I4YQ11;  A0A7I4XWS3;  A0A7I5E7R8;  A0A7I4Y9W7;  A0A7I4Z5T7;  A0A7I4YJF1;  A0A7I4YI94;  A0A7I4Z633;  A0A7I4Z1I0;  A0A7I4YHD0 | Down | 0.0028 |
| Biosynthesis of amino acids | Transket_pyr domain-containing protein；  Glutamine synthetase；  Serine hydroxymethyl transferase；  Threonine ammonia-lyase；  Isocitrate dehydrogenase [NADP]；  Phosphopyruvate hydratase；  Pyruvate kinase；  Phosphoglycerate kinase；  Fructose-bisphosphate aldolase | A0A7I4YJF1;  A0A7I4YU77;  A0A0N4W4W6;  A0A7I5E865;  A0A7I4Z2S6;  U6PW36;  A0A7I4YQ11;  A0A7I4Z1I0;  A0A7I4Y9W7 | Down | 0.003 |
| Citrate cycle (TCA cycle) | Dihydrolipoyl dehydrogenase；  Pyruvate dehydrogenase E1 component subunit beta；  Isocitrate dehydrogenase [NADP]；  Fumarate hydratase；  Acetyltransferase component of pyruvate dehydrogenase complex；  Succinate dehydrogenase flavoprotein subunit, mitochondrial；  Oxoglutarate dehydrogenase (succinyl-transferring) | A0A7I4YM01;  A0A6F7PVQ8;  A0A7I4Z2S6;  A0A6F7PEZ2;  A0A7I5EBZ2;  A0A7I4YZY5;  A0A7I4YHD0 | Down | 0.011 |
| Methane metabolism | Phosphopyruvate hydratase；  Serine hydroxymethyl transferase；  Fructose-bisphosphatase；  Fructose-bisphosphate aldolase | U6PW36;  A0A0N4W4W6;  A0A7I4Z5T7;  A0A7I4Y9W7 | Down | 0.019 |
| Glycine, serine and threonine metabolism | Serine hydroxymethyl transferase；  Threonine ammonia-lyase；  Dihydrolipoyl dehydrogenase；  Glycine cleavage system P protein | A0A0N4W4W6;  A0A7I5E865;  A0A7I4YM01;  A0A7I4Y9K3 | Down | 0.019 |
| Pentose phosphate pathway | Transket_pyr domain-containing protein；  Fructose-bisphosphatase；  Fructose-bisphosphate aldolase；  Glucose-6-phosphate isomerase | A0A7I4YJF1;  A0A7I4Z5T7;  A0A7I4Y9W7;  A0A7I4XWS3 | Down | 0.026 |
| HIF-1 signaling pathway | Pyruvate dehydrogenase E1 component subunit beta；  Phosphopyruvate hydratase；  40S ribosomal protein S6 | A0A6F7PVQ8;  U6PW36;  A0A7I4XVS6 | Down | 0.039 |
| RNA degradation | Phosphopyruvate hydratase；  Heat shock protein 60；  Stress-70 protein, mitochondrial | U6PW36;  A0A7I4YD57;  A0A3P7V1I6 | Down | 0.039 |
| Pyruvate metabolism | Pyruvate kinase;  Dihydrolipoyl dehydrogenase;  Fumarate hydratase;  Acetyltransferase component of pyruvate dehydrogenase complex;  Pyruvate dehydrogenase E1 component subunit beta;  Malic enzyme | A0A7I4YQ11;  A0A7I4YM01;  A0A6F7PEZ2;  A0A7I5EBZ2;  A0A6F7PVQ8;  A0A7I4Y809 | Down | 0.046 |
