# Supplementary material for: Comparative proteomics analysis of adult Haemonchus contortus isolates from Ovis ammon
Source: Front Cell Infect Microbiol. 2023 Mar 16;13:1087210. doi: 10.3389/fcimb.2023.1087210 (PMC10061303; doi:10.3389/fcimb.2023.1087210)
Supplement: Supplementary file 3 [file Table_2.docx]

Supplementary Table2. All-regulated DEPs of [*Haemonchus contortus*](https://www.uniprot.org/taxonomy/6289) in 2-vs-3 group

| Category_  Name | Description OS=  [*Haemonchus contortus*](https://www.uniprot.org/taxonomy/6289) | ProteinIDs | Regulated-Stage | Fisher’s exact test p value |
| --- | --- | --- | --- | --- |
| Protein digestion and absorption | Intestinal prolyl carboxypeptidase 2;  Intestinal prolyl carboxypeptidase 1;  Intestinal prolyl carboxypeptidase 2 | A5CG77;  A5CG76;  A0A7I4YFP2 | up | 0.009 |
| Renin-angiotensin system | Intestinal prolyl carboxypeptidase 2;  Intestinal prolyl carboxypeptidase 1;  Intestinal prolyl carboxypeptidase 2 | A5CG77;  A5CG76;  A0A7I4YFP2 | up | 0.015 |
| Biosynthesis of secondary metabolites | L-lactate dehydrogenase;  Pyruvate dehydrogenase E1 component subunit alpha; Aminomethyltransferase;  Alpha-1,4 glucan phosphorylase;  Isocitrate dehydrogenase [NADP];  Phosphoglucomutase  Enoyl-CoA hydratase;  Succinate--CoA ligase subunit beta, mitochondrial;  ATP-citrate synthase;  Succinate dehydrogenase iron-sulfur subunit, mitochondrial;  Cystathionine gamma-lyase;  Ornithine aminotransferase;  ATP-dependent 6-phosphofructokinase | A0A7I4Y0B7;  A0A7I4Y152;  A0A7I4YTF8;  A0A0N4WWC4;  A0A7I4Z2S6;  A0A7I4Y2T4;  A0A7I4XU77;  A0A7I5E822;  A0A7I5E8E4;  W6NKM1;  A0A7I4Y5R0;  A0A6F7P322;  A0A6F7PVV4 | up | 0.015 |
| Carbon fixation pathways in prokaryotes | Formate--tetrahydrofolate ligase;  Isocitrate dehydrogenase [NADP];  ATP-citrate synthase | A0A7I4Y292; A0A7I4Z2S6;  A0A7I5E8E4 | up | 0.025 |
| Glucagon signaling pathway | L-lactate dehydrogenase;  Pyruvate dehydrogenase E1 component subunit alpha;  Alpha-1,4 glucan phosphorylase | A0A7I4Y0B7;  A0A7I4Y152;  A0A0N4WWC4 | up | 0.032 |
| One carbon pool by folate | Aminomethyltransferase;  Formate--tetrahydrofolate ligase | A0A7I4YTF8;  A0A7I4Y292 | up | 0.037 |
| Alzheimer's disease | NADH dehydrogenase 1 alpha subcomplex subunit 13;  NADH dehydrogenase 1 alpha subcomplex subunit 5;  Succinate dehydrogenase iron-sulfur subunit, mitochondrial; NADH dehydrogenase flavoprotein 1, mitochondrial;  Calcium-transporting ATPase;  NADH dehydrogenase iron-sulfur protein 3, mitochondrial | A0A0N4WKE0;  A0A7I4YQ96;  W6NKM1;  A0A7I4Z8Z3;  A0A7I4Y9D1;  W6NH01 | up | 0.04 |
| Non-alcoholic fatty liver disease (NAFLD) | NADH dehydrogenase 1 alpha subcomplex subunit 5;  NADH dehydrogenase 1 alpha subcomplex subunit 13;  NADH dehydrogenase flavoprotein 1, mitochondrial;  Succinate dehydrogenase iron-sulfur subunit, mitochondrial;  NADH dehydrogenase iron-sulfur protein 3, mitochondrial | A0A7I4YQ96;  A0A0N4WKE0;  A0A7I4Z8Z3;  W6NKM1;  W6NH01 | up | 0.04 |
| Metabolic pathways | L-lactate dehydrogenase;  Pyruvate dehydrogenase E1 component subunit alpha;  NADH dehydrogenase iron-sulfur protein 3, mitochondrial; Aminomethyl transferase;  NADH dehydrogenase flavoprotein 1, mitochondrial;  Propionyl-CoA carboxylase beta chain, mitochondrial;  NADH dehydrogenase 1 alpha subcomplex subunit 13;  Alpha-1,4 glucan phosphorylase;  NADH dehydrogenase 1 alpha subcomplex subunit 5;  Formate--tetrahydrofolate ligase;  Isocitrate dehydrogenase [NADP];  Enoyl-CoA hydratase;  Phosphoglucomutase;  Succinate--CoA ligase subunit beta, mitochondrial;  ATP-citrate synthase;  Succinate dehydrogenase iron-sulfur subunit, mitochondrial; Adenylate kinase isoenzyme 1;  Cystathionine gamma-lyase;  Ornithine aminotransferase;  ATP-dependent 6-phosphofructokinase | A0A7I4Y0B7;  A0A7I4Y152;  W6NH01;  A0A7I4YTF8;  A0A7I4Z8Z3;  A0A7I5EDP0;  A0A0N4WKE0;  A0A0N4WWC4;  A0A7I4YQ96;  A0A7I4Y292;  A0A7I4Z2S6;  A0A7I4XU77;  A0A7I4Y2T4;  A0A7I5E822;  A0A7I5E8E4;  W6NKM1;  A0A7I4Z188;  A0A7I4Y5R0;  A0A6F7P322;  A0A6F7PVV4 |  | 0.04 |
| Carbon  metabolism | Transket_pyr domain-containing protein；  Serine hydroxymethyl transferase；  Dihydrolipoyl dehydrogenase；  Probable methylmalonate-semialdehyde dehydrogenase, mitochondrial；  Pyruvate dehydrogenase E1 component subunit beta；  Aconitate hydratase, mitochondrial；  Fumarate hydratase；  Glutamate dehydrogenase；  Acetyltransferase component of pyruvate dehydrogenase complex；  Threonine ammonia-lyase；  Phosphopyruvate hydratase；  Pyruvate kinase；  Glucose-6-phosphate isomerase； | A0A7I4YJF1;  A0A0N4W4W6;  A0A7I4YM01;  A0A7I4Y6H9;  U6PAC8;  A0A6F7PVQ8;  A0A7I5E9A3;  A0A6F7PEZ2;  A0A7I4YI94;  A0A7I5EBZ2;  A0A7I5E865;  U6PW36;  A0A7I4YQ11;  A0A7I4XWS3;  A0A7I4Y9W7 | down | 0.0016 |
| Longevity regulating pathway - worm | Stress-70 protein, mitochondrial；  Heat shock protein 60；  Superoxide dismutase | A0A3P7V1I6;  A0A7I4YD57;  A0A3P7YJP8 | down | 0.009 |
| Legionellosis | Elongation factor 1-alpha；  Vesicle-fusing ATPase；  Elongation factor 1-gamma；  Heat shock protein 60；  Heat shock protein 70 | A0A7I4XYJ2;  A0A0N4WNI2;  A0A7I5ECW3;  A0A7I4YD57;  A0A7I4YQ65 | down | 0.018 |
| Glycolysis / Gluconeogenesis | Pyruvate kinase；  Dihydrolipoyl dehydrogenase；  Pyruvate dehydrogenase E1 component subunit beta；  Acetyltransferase component of pyruvate dehydrogenase complex； Phosphopyruvate hydratase；  Glucose-6-phosphate isomerase；  Fructose-bisphosphate aldolase | A0A7I4YQ11;  A0A7I4YM01;  A0A6F7PVQ8;  A0A7I5EBZ2;  U6PW36;  A0A7I4XWS3;  A0A7I4Y9W7 | down | 0.019 |
| Protein processing in endoplasmic reticulum | Heat shock protein 70；  Vesicle-fusing ATPase；  Protein disulfide-isomerase；  Heat shock protein 70；  Calreticulin；  Protein disulfide-isomerase | A0A7I4YHA9;  A0A0N4WNI2;  A0A7I5E638;  A0A7I4YQ65;  A0A7I5EC72;  A0A7I4Z4X8 | down | 0.019 |
| Biosynthesis of antibiotics | Serine hydroxymethyl transferase;  Dihydrolipoyl dehydrogenase;  Pyruvate dehydrogenase E1 component subunit beta;  Aconitate hydratase, mitochondrial;  Fructose-bisphosphate aldolase;  Fumarate hydratase;  Acetyltransferase component of pyruvate dehydrogenase complex; Threonine ammonia-lyase;  Phosphopyruvate hydratase;  Pyruvate kinase;  Glucose-6-phosphate isomerase;  Nucleoside diphosphate kinase;  Transket_pyr domain-containing protein | A0A0N4W4W6;  A0A7I4YM01;  A0A6F7PVQ8;  A0A7I5E9A3;  A0A7I4Y9W7;  A0A6F7PEZ2;  A0A7I5EBZ2;  A0A7I5E865;  U6PW36;  A0A7I4YQ11;  A0A7I4XWS3;  A0A7I4XSS8;  A0A7I4YJF1 | down | 0.029 |
| RNA degradation | Phosphopyruvate hydratase;  Heat shock protein 60;  Stress-70 protein, mitochondrial | U6PW36;  A0A7I4YD57;  A0A3P7V1I6 | down | 0.042 |
| Biosynthesis of amino acids | Transket_pyr domain-containing protein;  Serine hydroxymethyl transferase;  Aconitate hydratase, mitochondrial;  Threonine ammonia-lyase;  Phosphopyruvate hydratase;  Pyruvate kinase;  Fructose-bisphosphate aldolase | A0A7I4YJF1;  A0A0N4W4W6;  A0A7I5E9A3;  A0A7I5E865;  U6PW36;  A0A7I4YQ11;  A0A7I4Y9W7 | down | 0.045 |
