# Supplementary material for: Comparative proteomics analysis of adult Haemonchus contortus isolates from Ovis ammon
Source: Front Cell Infect Microbiol. 2023 Mar 16;13:1087210. doi: 10.3389/fcimb.2023.1087210 (PMC10061303; doi:10.3389/fcimb.2023.1087210)
Supplement: Supplementary file 4 [file Table_3.docx]

Supplementary Table3. All-regulated DEPs of [*Haemonchus contortus*](https://www.uniprot.org/taxonomy/6289) in 2-vs-1 group

| Category_  Name | Description OS=  [*Haemonchus contortus*](https://www.uniprot.org/taxonomy/6289) | ProteinIDs | Regulated-Stage | Fisher's exact test p value |
| --- | --- | --- | --- | --- |
| Microbial  metabolism  in diverse  environments | Succinate dehydrogenase flavoprotein subunit, mitochondrial；  Serine hydroxymethyl transferase；  Dihydrolipoyl dehydrogenase；  Glutamine synthetase；  Pyruvate dehydrogenase E1 component subunit beta；  Succinate--CoA ligase [ADP-forming] subunit beta, mitochondrial； Isocitrate dehydrogenase [NADP]；  ATP-citrate synthase；  Malic enzyme；  Transket_pyr domain-containing protein；  Formate--tetrahydrofolate ligase；  Oxoglutarate dehydrogenase (succinyl-transferring)；  Fructose-bisphosphatase；  Phosphoglycerate kinase | A0A7I4YZY5;  A0A0N4W4W6;  A0A7I4YM01;  A0A7I4YU77;  A0A6F7PVQ8;  A0A7I5E822;  A0A7I4Z2S6;  A0A7I5E8E4;  A0A7I4Y809;  A0A7I4YJF1;  A0A7I4Y292;  A0A7I4YHD0;  A0A7I4Z5T7;  A0A7I4Z1I0 | UP | 0.0007 |
| Carbon metabolism | Succinate dehydrogenase flavoprotein subunit, mitochondrial；  Transket_pyr domain-containing protein；  Serine hydroxymethyl transferase；  Dihydrolipoyl dehydrogenase；  Pyruvate dehydrogenase E1 component subunit beta；  Succinate--CoA ligase [ADP-forming] subunit beta, mitochondrial；  Isocitrate dehydrogenase [NADP]；  Glycine cleavage system P protein；  Formate--tetrahydrofolate ligase；  Oxoglutarate dehydrogenase (succinyl-transferring)；  Fructose-bisphosphatase；  Phosphoglycerate kinase | A0A7I4YZY5;  A0A7I4YJF1;  A0A0N4W4W6;  A0A7I4YM01;  A0A6F7PVQ8;  A0A7I5E822;  A0A7I4Z2S6;  A0A7I4Y9K3;  A0A7I4Y809;  A0A7I4Y292;  A0A7I4YHD0;  A0A7I4Z5T7;  A0A7I4Z1I0 | UP | 0.001 |
| Citrate cycle (TCA cycle) | Dihydrolipoyl dehydrogenase；  Pyruvate dehydrogenase E1 component subunit beta；  Succinate--CoA ligase [ADP-forming] subunit beta, mitochondrial；  Isocitrate dehydrogenase [NADP]；  ATP-citrate synthase；  Succinate dehydrogenase flavoprotein subunit, mitochondrial；  Oxoglutarate dehydrogenase (succinyl-transferring) | A0A7I4YM01;  A0A6F7PVQ8;  A0A7I5E822;  A0A7I4Z2S6;  A0A7I5E8E4;  A0A7I4YZY5;  A0A7I4YHD0 | UP | 0.0015 |
| Metabolic pathways | Glycine cleavage system P protein；  Aminotran_1_2 domain-containing protein；  ATP synthase subunit alpha；  Serine hydroxymethyl transferase；  Dihydrolipoyl dehydrogenase；  NADH dehydrogenase [ubiquinone] flavoprotein 1, mitochondrial；  Glutamine synthetase；  Propionyl-CoA carboxylase beta chain, mitochondrial；  NADH dehydrogenase 1 alpha subcomplex subunit 13；  Propionyl-CoA carboxylase alpha chain, mitochondrial；  Succinate dehydrogenase flavoprotein subunit, mitochondrial；  Adenosyl homocysteinase；  Pyruvate dehydrogenase E1 component subunit beta；  Formate--tetrahydrofolate ligase；  Isocitrate dehydrogenase [NADP]；  Malic enzyme；  Fructose-bisphosphatase；  Transket_pyr domain-containing protein；  Succinate--CoA ligase [ADP-forming] subunit beta, mitochondrial；  ATP-citrate synthase；  ATP synthase subunit beta；  Phosphoglycerate kinase；  Oxoglutarate dehydrogenase (succinyl-transferring) | A0A7I4Y9K3;  A0A7I4YNV2;  A0A7I4Y004;  A0A0N4W4W6;  A0A7I4YM01;  A0A7I4Z8Z3;  A0A7I4YU77;  A0A7I5EDP0;  A0A0N4WKE0;  A0A7I5E751;  A0A7I4YZY5;  A0A7I4XY18;  A0A6F7PVQ8;  A0A7I4Y292;  A0A7I4Z2S6;  A0A7I4Y809;  A0A7I4Z5T7;  A0A7I4YJF1;  A0A7I5E822;  A0A7I5E8E4;  A0A7I4Z633;  A0A7I4Z1I0;  A0A7I4YHD0 | UP | 0.003 |
| Biosynthesis of antibiotics | Succinate dehydrogenase flavoprotein subunit, mitochondrial；  Serine hydroxymethyl transferase；  Dihydrolipoyl dehydrogenase；  Pyruvate dehydrogenase E1 component subunit beta；  Succinate--CoA ligase subunit beta, mitochondrial；  Isocitrate dehydrogenase [NADP]；  Phosphoglycerate kinase；  ATP-citrate synthase；  Glycine cleavage system P protein；  Transket_pyr domain-containing protein；  Oxoglutarate dehydrogenase (succinyl-transferring)； Fructose-bisphosphatase | A0A7I4YZY5;  A0A0N4W4W6;  A0A7I4YM01;  A0A6F7PVQ8;  A0A7I5E822;  A0A7I4Z2S6;  A0A7I4Z1I0;  A0A7I5E8E4;  A0A7I4Y9K3;  A0A7I4YJF1;  A0A7I4YHD0;  A0A7I4Z5T7 | UP | 0.0041 |
| Carbon fixation pathways  in prokaryotes | Formate--tetrahydrofolate ligase；  Isocitrate dehydrogenase [NADP]；  ATP-citrate synthase | A0A7I4Y292;  A0A7I4Z2S6;  A0A7I5E8E4 | UP | 0.0079 |
| Glyoxylate and dicarboxylate metabolism | Glutamine synthetase；  Serine hydroxymethyl transferase；  Dihydrolipoyl dehydrogenase；  Glycine cleavage system P protein | A0A7I4YU77;  A0A0N4W4W6;  A0A7I4YM01;  A0A7I4Y9K3 | UP | 0.0086 |
| One carbon pool by folate | Formate--tetrahydrofolate ligase；  Serine hydroxymethyl transferase | A0A7I4Y292;  A0A0N4W4W6 | UP | 0.0129 |
| Protein processing in endoplasmic reticulum | Heat shock protein 70；  Protein disulfide-isomerase；  Heat shock protein 70；  Calreticulin；  Protein disulfide-isomerase | A0A7I4YHA9;  A0A7I5E638;  A0A7I4YQ65;  A0A7I5EC72;  A0A7I4Z4X8 | Down | 0.0044 |
| Arginine biosynthesis | Aspartate aminotransferase；  Glutamate dehydrogenase；  Alanine transaminase | A0A7I4YXS0;  A0A7I4YI94;  A0A7I4XTD0 | Down | 0.0045 |
| Estrogen signaling pathway | Peptidylprolyl isomerase；  Heat shock protein 70 | A0A6F7PVL7;  A0A7I4YQ65 | Down | 0.0069 |
| Longevity regulating pathway - multiple species | Heat shock protein 70；  Superoxide dismutase | A0A7I4YQ65;  A0A3P7YJP8 | Down | 0.0069 |
| Longevity regulating pathway - worm | Heat shock protein 60；  Superoxide dismutase | A0A7I4YD57;  A0A3P7YJP8 | Down | 0.0206 |
| Alanine, aspartate and glutamate metabolism | Alanine transaminase；  Aspartate aminotransferase；  Glutamate dehydrogenase | A0A7I4XTD0;  A0A7I4YXS0;  A0A7I4YI94 | Down | 0.0206 |
